# Navigating the Maze: Exploring Non-Oncological Complexities in Non-Small-Cell Lung Cancer

**DOI:** 10.3390/cancers16101903

**Published:** 2024-05-16

**Authors:** Angela-Ștefania Marghescu, Silviu Vlăsceanu, Mădălina Preda, Mirela Țigău, Ștefan Dumitrache-Rujinski, Diana Gabriela Leonte, Elena Doina Măgheran, Adrian Tudor, Ioana Anca Bădărău, Livia Georgescu, Mariana Costache

**Affiliations:** 1 Pathology Department, Faculty of Medicine, Carol Davila University of Medicine and Pharmacy, 020021 Bucharest, Romania; angela.varban@drd.umfcd.ro (A.-Ș.M.); mariana.costache@umfcd.ro (M.C.); 2 Department of Research, Marius Nasta Institute of Pneumophthisiology, 050159 Bucharest, Romania; mirela.tigau@marius-nasta.ro (M.Ț.); livia.georgescu@marius-nasta.ro (L.G.); 3 Physiology Department, Faculty of Medicine, Carol Davila University of Medicine and Pharmacy, 020021 Bucharest, Romania; anca.badarau@umfcd.ro; 4 Department of Thoracic Surgery, Marius Nasta Institute of Pneumophthisiology, 050159 Bucharest, Romania; 5 Department of Microbiology, Parasitology and Virology, Faculty of Midwives and Nursing, “Carol Davila” University of Medicine and Pharmacy, 020021 Bucharest, Romania; 6 Clinical Laboratory of Medical Microbiology, Marius Nasta Institute of Pneumology, 050159 Bucharest, Romania; 7 Pulmonology Department, Faculty of Medicine, Carol Davila University of Medicine and Pharmacy, 020021 Bucharest, Romania; stefan.dumitrache@umfcd.ro; 8 Pulmonology Department, Marius Nasta Institute of Pneumophthisiology, 050159 Bucharest, Romania; 9 Pathology Department, Marius Nasta Institute of Pneumophthisiology, 050159 Bucharest, Romania; diana.leonte@personalgenetics.ro (D.G.L.); elena.magheran@marius-nasta.ro (E.D.M.); adrian.tudor@marius-nasta.ro (A.T.); 10 Pathology Department, University Emergency Hospital Bucharest, 050098 Bucharest, Romania

**Keywords:** lung cancer, NSCLC, coexisting lesions

## Abstract

**Simple Summary:**

Pulmonary oncological issues are a significant public health concern, often complicated by the presence of other pulmonary conditions, which can complicate diagnosis, staging, and treatment decisions. To explore this complexity, we conducted a retrospective study at the Marius Nasta Institute of Pneumophthisiology in Bucharest, Romania, focusing on patients diagnosed with carcinoid and/or non-small-cell lung carcinoma and coexisting pathologies. Patients with both malignant and nonmalignant lesions shared similar demographic and exposure profiles with those presenting only malignant lesions. Synchronous coexisting lesions may complicate the diagnosis and staging of lung cancer.

**Abstract:**

Pulmonary oncological pathologies are an important public health problem and the association with other pulmonary lesions may pose difficulties in diagnosis and staging or require different treatment options. To address this complexity, we conducted a retrospective observational study at the Marius Nasta Institute of Pneumophthisiology, Bucharest, Romania. Our study focused on patients admitted in 2019 with non-small-cell lung carcinoma and associated pulmonary lesions identified through surgical resection specimens. Among the 314 included patients, multiple pulmonary nodules were observed on macroscopic examination, with 12% (N = 37) exhibiting nonmalignant etiologies upon microscopic examination. These findings underscore the challenge of preoperative staging. Patients with coexisting nonmalignant lesions were similar in age, smoking habits, and professional or environmental exposure by comparison with those who presented only malignant lesions. The presentation of coexisting malignant and nonmalignant lesions may pose difficulties in diagnosing and staging pulmonary cancer.

## 1. Introduction

Oncological pathologies pose a major public health problem, incurring high costs for both diagnosis and treatment. Worldwide, bronchopulmonary cancer ranks as the second most frequent neoplasia, with a staggering 2,206,771 cases reported in 2020 accompanied by the highest mortality among oncological patients, with 1,796,144 deaths recorded in the same year [1]. 

In 2020, a significant number of new lung cancer cases (12,122) were diagnosed in Romania, but our country also has a high incidence of other bronchopulmonary pathologies, such as tuberculosis [1,2]. Given this context, the diagnosis of conditions associated with lung cancer presents formidable challenges, profoundly impacting treatment decisions. 

To confirm malignancy, determine histological subtype, pinpoint the tumor’s origin, and guide further molecular investigations, the assessment of lung tumors by surgical pathologists is indispensable in the clinical diagnostic pathway [3,4]. Besides establishing the tumor histogenesis, the histopathological examination helps in establishing the tumor, node, metastasis (TNM) stage of the cancer. The importance of a clear and reliable diagnosis is emphasized by the fact that each of these considerations is vital in choosing a course of treatment [3]. Thus, a histopathological examination stands as a pivotal step in the diagnosis of lung cancer, currently representing the definitive investigation for confirming neoplasia. 

The diverse spectrum of lung cancer encompasses over 50 histomorphological subtypes [5]. Epithelial tumors of the lung are classified as non-small-cell lung carcinoma (NSCLC) and small-cell lung carcinoma (SCLC), given the different prognostic and therapeutical options [5]. Adenocarcinoma (ADC, 40–50% of cases) and squamous cell carcinoma (SqCC, 20–30% of cases) are the two most common histological subtypes of NSCLC, accounting for 80–85% of all cases of lung cancer [5].

For surgical pathologists, diagnosing patients with numerous lung tumors and other abnormalities might be difficult [6]. Distinguishing between independent primary lung malignancies and primary tumors with satellite lesions, intrapulmonary metastases, or other pathologies is imperative, as clinical management and prognostication differ between these entities [6].

While previous studies have focused on the oncological aspects of NSCLC, such as tumor biology, staging, and treatment modalities, few have explored the impact of concurrent non-malignant diseases. Few investigations have delved into the intricate interplay between NSCLC and these coexisting conditions, leaving a crucial aspect of the disease’s complexity and its management relatively unexplored. This aspect is of great importance, especially in countries with increased tuberculosis.

The objectives of this study were to ascertain the prevalence of lung cancer cases associated with other pulmonary lesions among patients diagnosed with lung cancer in 2019 at the Marius Nasta Institute of Pneumophthisiology, Bucharest, Romania, analyze their associations and localizations, and evaluate the impact of risk factors such as cigarette smoking and occupational exposure to respiratory carcinogens.

## 2. Materials and Methods

We conducted a retrospective observational study at the Marius Nasta Institute of Pneumophthisiology, Bucharest, Romania, focusing on patients diagnosed with pulmonary malignancy in 2019 based on surgical resection specimens. To identify eligible patients with lung cancer and associated lesions, we thoroughly searched the database of the Pneumology Institute and reviewed their electronic health records. For each patient included in the study, comprehensive data were recorded using a standardized form. All cases were anonymized and assigned identification codes for subsequent analysis, incorporating demographic, clinical, and paraclinical characteristics. 

Inclusion criteria encompassed patients aged 18 and above who underwent surgical resection and received a diagnosis of carcinoid (typical/atypical) and/or NSCLC at the Pathology Department of the Marius Nasta Institute of Pneumophthisiology. Additionally, patients with combined carcinomas featuring small-cell components were included. Hence, our study included patients with lung tumor types for which surgical resection is a viable therapeutic option such as carcinoids and NSCLC lesions. Exclusion criteria comprised patients under 18 years old, those with pathological diagnoses established outside our medical unit, individuals diagnosed with primary lung cancers other than carcinoid and NSCLC, lung metastases, benign pulmonary tumors, or non-neoplastic pulmonary diseases, as well as prisoners and individuals with inadequate mental health statuses.

Standard examination protocol for the surgical specimens begins with fixation in 10% buffered formalin. Significant tissue fragments are collected and processed using standard methods, with select cases undergoing additional special stainings, such as Ziehl–Neelsen staining following initial hematoxylin–eosin examination. Histology slides are examined in our Pathology Department using an Olympus BX46 microscope (Olympus, Tokio, Japan), and images are captured with an Olympus SC50 microscope camera.

From the cohort of included patients, cases featuring multiple coexisting lesions evident upon gross examination were identified, reflecting challenges in macroscopic diagnosis. 

Data management and analysis were performed using Statistical Product and Service Sciences version 22.0 (SPSS Inc., Chicago, IL, USA) after entry into Microsoft Excel 2021 (Microsoft 365) spreadsheets. Descriptive analyses were conducted, with continuous variables reported as mean ± SD or median [IQR]. Differences between categorical variables were assessed using the Mann–Whitney U test, with statistical significance set at a *p*-value < 0.05.

Before any investigation or procedure, patients provided informed consent upon admission to the Institute, consenting to participation in clinical studies. This study received approval from the Ethics Committee of the Marius Nasta Institute of Pneumophthisiology (approval code 7947/6 April 2022).

## 3. Results

In 2019, a total of 314 surgical resection samples were identified from patients diagnosed with non-small-cell lung cancer at the Pathology Department of Marius Nasta Institute of Pneumophthisiology, Bucharest, Romania. Among these cases, 12% (N = 37) presented diagnostic complexities due to the identification of multiple lesions during the macroscopic examination of the resection specimens.

The mean age among individuals diagnosed solely with lung cancer (N = 277) was 63.12 ± 9.4 years, while, in those with coexisting lesions, it was 60.62 ± 8.5 years, with no statistically significant difference observed (*p* = 0.134). Approximately three-quarters (75.67%) of cases with multiple lesions hailed from urban areas, and two-thirds (62%) were male.

Regarding cigarette-smoking habits, among patients diagnosed solely with NSCLC, 33.57% were active smokers (with an average of 42.98 pack-years), 31.40% were ex-smokers, and 35.03% were never-smokers. Among patients with coexisting lesions, 35.13% were active smokers (with an average of 41.15 pack-years), 40.54% were ex-smokers, and 24.33% were never-smokers; a history of smoking was present in 75.7% of these cases, consistent with the literature-reported data. A notable proportion (27%) of the patients with coexisting lesions reported occupational exposure to respiratory carcinogens, contrasting with 17.68% in NSCLC patients without associated lesions, although this difference lacked statistical significance (*p* = 0.17). 

**Figure 1:**
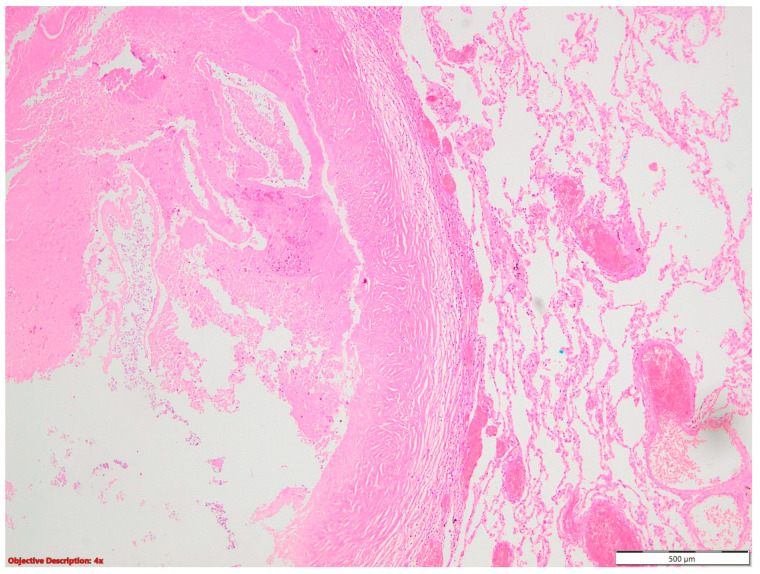
Fibronecrotic nodule (**left** side) in the lung parenchyma (**right** side); HE, 40×.

A histopathological examination of surgical resection specimens highlighted the presence of the following associations between NSCLC and other tumoral and nontumoral, and infectious and noninfectious pathological lesions, including nine cases of granulomatous inflammation, ten cases of fibronodular lesions, five cases of fibronecrotic nodules (Figure 1), two nodular calcifications, four instances of osseous/osteomedullary metaplasia, three cases of pneumoconiosis, two benign tumors, four lung infarctions, one instance of aspiration pneumonia, and one meningothelial-like nodule (Table 1).

**Figure 2:**
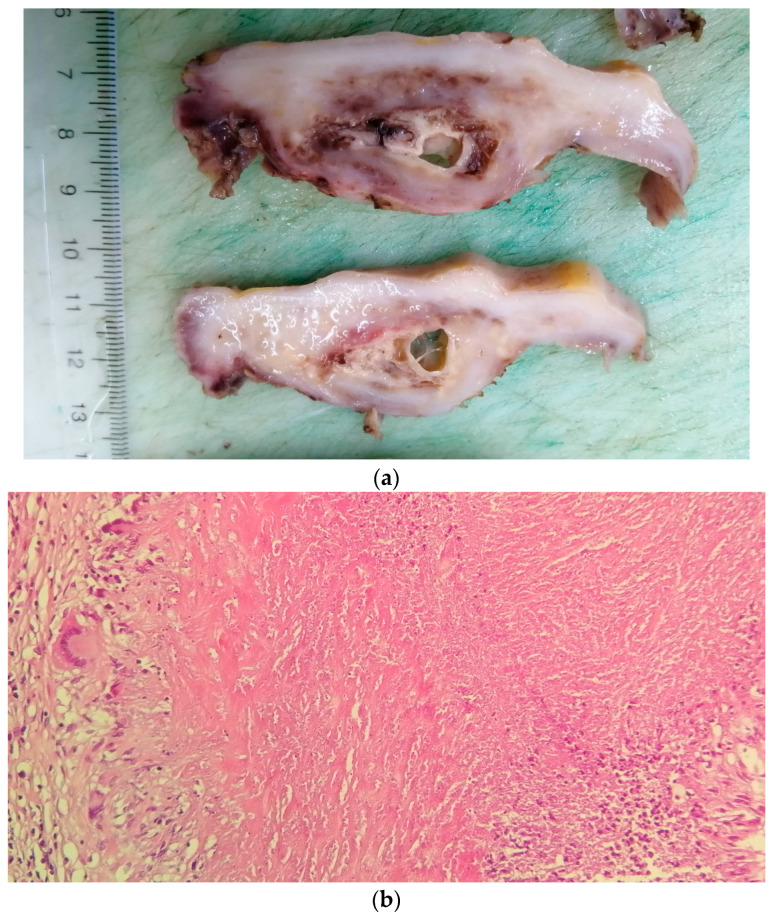

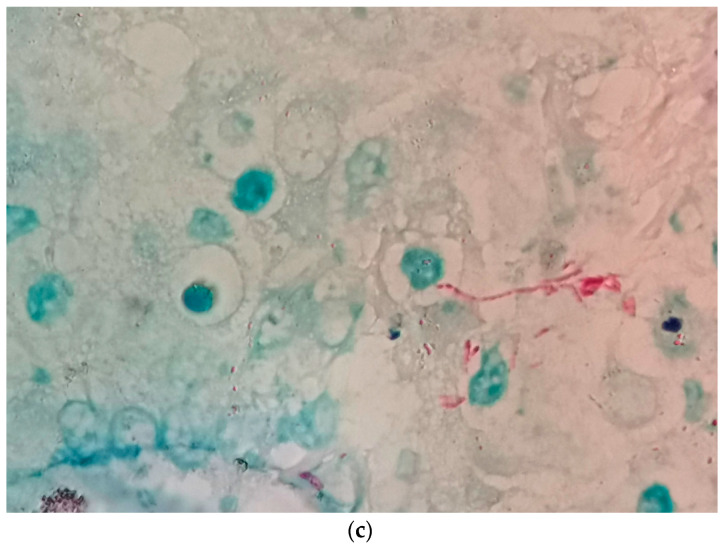
Tuberculosis: (**a**) gross examination (fibronecrotic lesion—caseous necrosis); (**b**) microscopic examination (necrotizing granuloma including Langhans giant cells and epithelioid histiocytes; HE, 400×); and (**c**) microscopic examination (BAAR; Ziehl Neelsen, 1000×).

**Figure 3:**
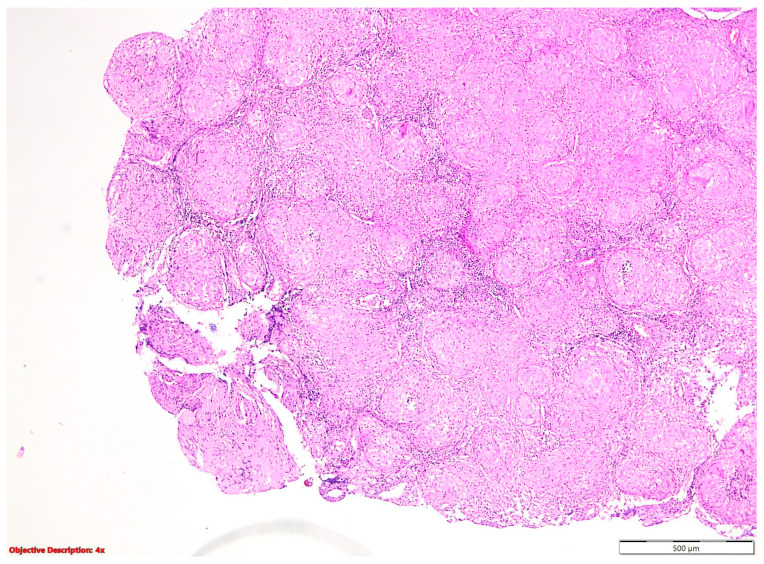
Sarcoidosis—well-formed noncaseating epithelioid granulomas; HE, 40×.

In the nine cases of NSCLC associated with granulomatous lesions, five of them were represented by active tuberculosis (Figure 2) and four by sarcoidosis (Figure 3).

**Figure 4:**
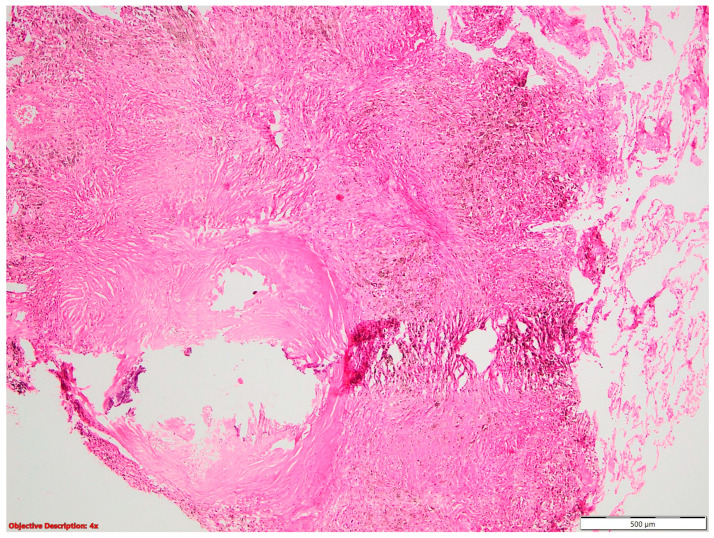
Fibronodular lesion (**left** side) in the lung parenchyma (**right** side); HE, 40×.

**Figure 5:**
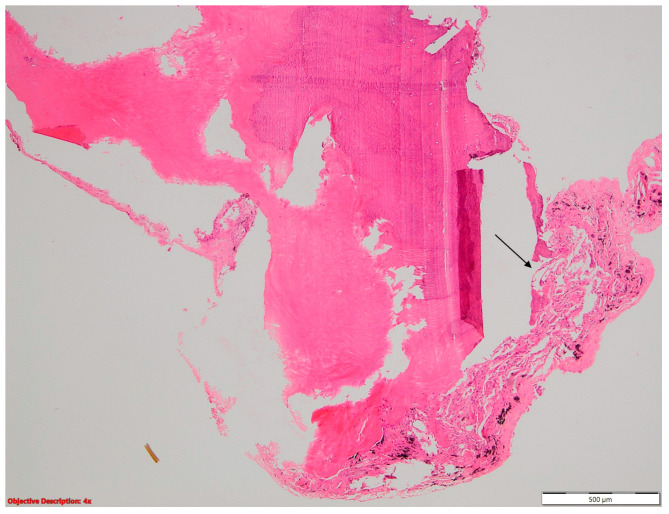
Osseous metaplasia in the lung (→: lung parenchima); HE, 40×.

Regarding the high prevalence of *Mycobacterium tuberculosis* in our country [2,7], all or part of the fibronodular lesions of the lung parenchyma (Figure 4), nodular calcification, and osseous/osteomedullary metaplasia (Figure 5) could represent sequelae of tuberculosis.

**Figure 6:**
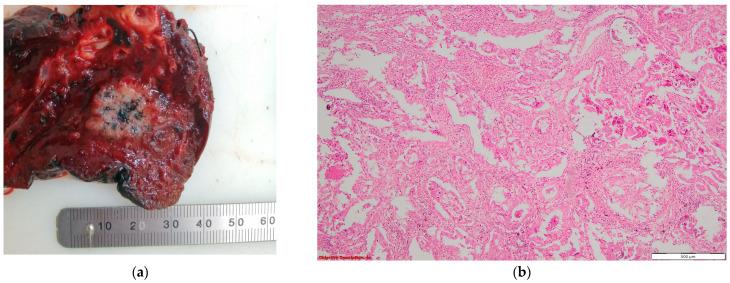
Lung adenocarcinoma: (**a**) gross examination (white tumor with polycyclic contour and anthracotic deposits); and (**b**) microscopic examination (tumoral glands that replace the normal lung parenchyma); HE, 40×.

**Figure 7:**
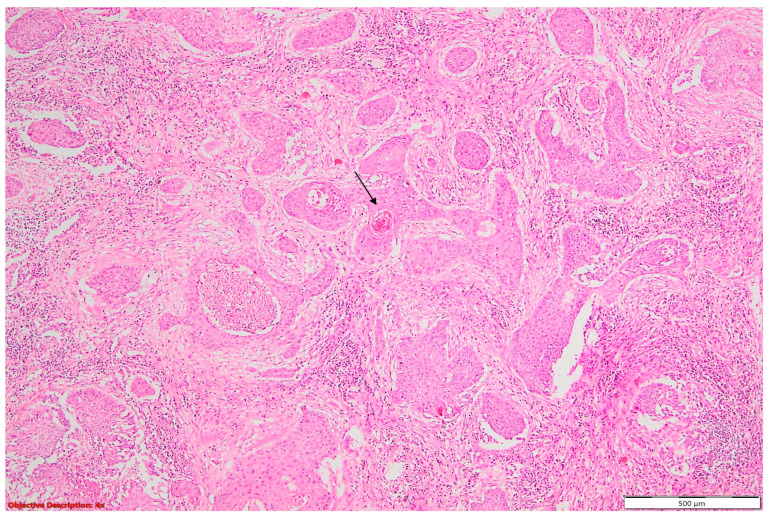
Lung squamous cell carcinoma (large polygonal cells with infiltrative pattern of growth; keratinization is evident →); HE, 40×.

**Figure 8:**
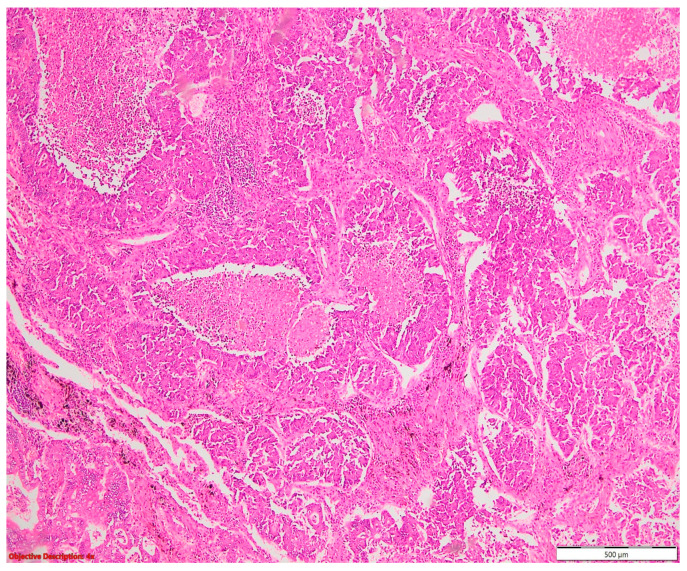
Large-cell neuroendocrine cell carcinoma with abundant tumoral necrosis; HE, 40×.

Two of the specimens with fibronodular lesions were associated with multiple NSCLC tumors of different histopathological subtypes: one case presented with adenocarcinoma (one tumor—Figure 6) and a squamous cell tumor (one tumor—Figure 7) and another case presented with adenocarcinoma (one tumor) and large-cell neuroendocrine tumors (two tumors—Figure 8).

The histopathological diagnosis identified an association between bronchopulmonary cancer and pneumoconiosis in three cases, all of them mild forms. Two of them were associated with adenocarcinoma and one with pleomorphic carcinoma having an adenocarcinoma component.

**Figure 9:**
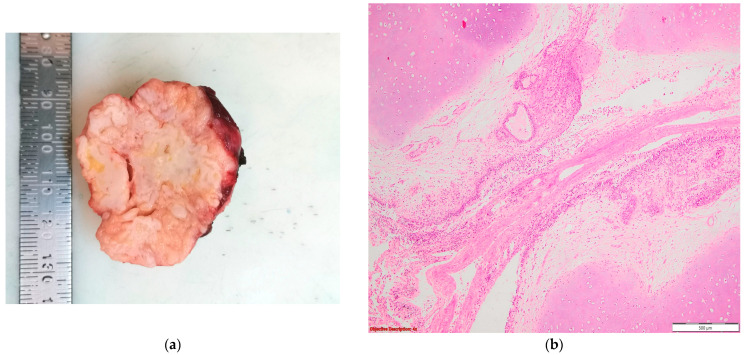
Lung hamartoma: (**a**) gross examination (multilobulated, well-circumscribed, and firm nodule); (**b**) microscopic examination (lobules of mature cartilage and clefts of entrapped respiratory epithelial cells); HE, 40×.

In the cases of benign tumors associated with NSCLC, the lung cancer presented as adenocarcinoma, and the coexisting lesion was represented in both cases by hamartoma (Figure 9). Although the number of cases reported with these associations is low, we consider that this observation can represent a hypothesis for future studies.

In our study, we uncovered an association between NSCLC and lung infarction, identifying four patients with this combination. However, upon microscopic examination, lymphovascular invasion was observed in only two of these cases.

**Figure 10:**
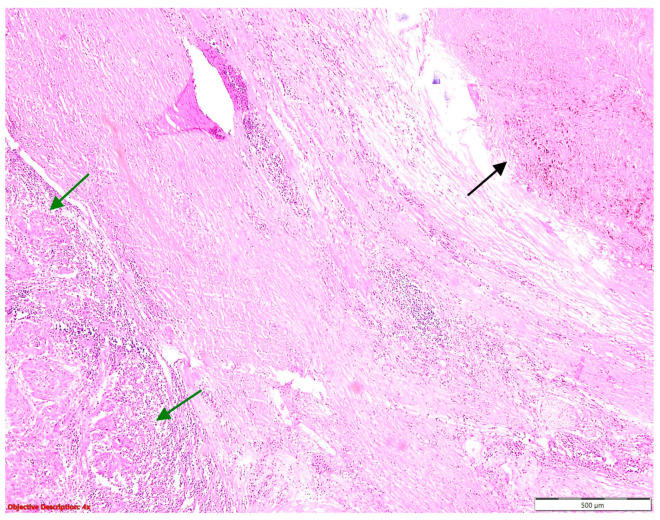
Coexisting lesions: lung carcinoma (**→**) and fibronodular lesion (→); HE, 40×.

When examining the localization of coexisting lesions, a fascinating pattern emerged: a majority of cases (43%) showcased lesions within the same lobe (N = 16) (Figure 10). This intriguing observation raises questions about the accuracy of tumor staging, as lesions within the same lobe may suggest T3 tumor extension, potentially leading to misclassification and subsequent treatment decisions. Furthermore, 27% of cases exhibited lesions concurrently in both the same lobe and lymph nodes. This finding underscores the complexity of lesion distribution and highlights the need for meticulous nodal staging to accurately assess disease spread. In comparison, an additional 27% presented coexisting lesions spanning across different lobes (N = 10), posing challenges in determining the extent of tumor involvement and guiding surgical resection boundaries. Remarkably, only one case featured multiple lesions dispersed across distinct lung lobes and lymph nodes, underscoring the rarity and diagnostic dilemma posed by such multifocal presentations. These findings underscore the importance of the careful consideration of lesion localization in NSCLC staging to minimize staging errors, inform treatment decisions, and improve patient outcomes.

## 4. Discussion

Our study delves into the intricate web of non-oncological factors influencing the diagnosis and staging of NSCLC. Through a comprehensive analysis, we showed significant insights into how concurrent non-malignant diseases intersect with NSCLC, challenging conventional diagnostic and staging protocols. By shedding light on these complexities, our findings underscore the imperative for a holistic approach to NSCLC management, one that integrates a thorough evaluation of both oncological and non-oncological aspects. Such an approach promises to refine current diagnostic and staging protocols, ensuring more accurate assessments and tailored treatment strategies for patients navigating the maze of the most common types of lung cancer. 

By integrating an understanding of non-oncological complexities into diagnostic protocols for NSCLC, clinicians can adopt a more nuanced approach to patient assessment. This entails not only focusing on tumor characteristics but also considering how concurrent non-malignant diseases may influence symptom presentation, radiographic interpretation, and laboratory findings. For instance, the presence of comorbid conditions such as chronic obstructive pulmonary disease (COPD) or cardiovascular disease can complicate the interpretation of imaging studies and pulmonary function tests, potentially leading to a misdiagnosis or delayed diagnosis of NSCLC. By incorporating a thorough evaluation of these non-oncological factors into diagnostic algorithms, clinicians can enhance the accuracy of NSCLC diagnosis, ensuring the timely initiation of appropriate treatment strategies.

Our patients exhibited a median age at diagnosis notably below the global median age of 71 years reported for lung cancer [8], suggesting potential demographic variations or underlying factors influencing disease onset.

Each patient was carefully selected to benefit from the thoracic surgery in a tertiary center. The treatment was performed in a University Surgery Clinic and it was maximal, optimal, and individualized, respecting the applicable international guidelines.

The influence of smoking on lung cancer development is well-established, with various factors affecting the dosage of smoke constituents ingested beyond just the cigarette itself. Factors such as inhalation intensity, the presence of filters, the length of time the smoke is allowed to cool before inhalation, and smoking patterns significantly impact individual exposure [9]. Despite modern cigarettes containing reduced nicotine and tar levels, smokers tend to compensate by intensifying their smoking habits, driven by nicotine dependence [9]. Consequently, estimates from smoking machines may grossly underestimate actual exposure levels. Furthermore, the prevalence of low-yield filtered cigarettes may contribute to the rising incidence of lung adenocarcinoma [9]. Notably, lung cancer affects a substantial proportion of never-smokers, accounting for approximately 25% of cases [10], mirroring our study’s findings where 27% of patients in the lung cancer group were non-smokers. These insights underscore the complex interplay between smoking habits, tobacco product characteristics, and lung cancer risk, highlighting the need for comprehensive strategies to mitigate disease burden among both smokers and non-smokers alike.

Expanding the spectrum of lung cancer risk factors, exposure to second-hand smoke, radon, and occupational toxins emerge as significant contributors. Additionally, the inhalation of cooking oil vapors and indoor coal burning further elevate one’s susceptibility to the disease [11]. Beyond environmental factors, infections such as HPV and *Mycobacterium tuberculosis*, along with hormonal, dietary, and metabolic variables, including diabetes mellitus, have also been implicated in the multifaceted etiology of lung cancer [10,12,13]. These diverse risk factors underscore the intricate interplay of genetic, environmental, and lifestyle influences in shaping lung cancer incidence and underscore the necessity for comprehensive preventive strategies.

The designation of “never-smokers”, which encompasses lifetime non-smokers, denotes individuals who have abstained from smoking more than 100 cigarettes throughout their entire lives [9]. Lung cancer cases among never-smokers exhibit distinctive clinical, pathological, and molecular features, with a higher prevalence observed among females and adenocarcinoma emerging as the predominant histological subtype. Moreover, molecular alterations in never-smokers’ lung cancer exhibit a distinct profile compared to those in smokers [10].

Across numerous studies, survival outcomes have consistently favored non-smoker patients with lung cancer over smokers [11]. Interestingly, our observations revealed a higher proportion of active and ex-smokers among patients diagnosed with lung cancer associated with coexisting lesions compared to those solely diagnosed with lung cancer. This discrepancy underscores the complex interplay between smoking history, disease progression, and the presence of coexisting lesions, warranting a further investigation into its implications on patient outcomes and treatment strategies.

Noncaseating granulomatous inflammation, with its potential to manifest as sarcoidosis, can impact multiple organs, contributing to a spectrum of clinical presentations [14]. Interestingly, sarcoidosis not only predisposes individuals to lung cancer but also shares clinical resemblances with viral, neoplastic, and other granulomatous conditions [15]. Although sarcoidosis and primary lung cancer infrequently coexist, their co-occurrence presents diagnostic challenges due to overlapping clinical features and histopathological findings [14]. This intricate relationship underscores the importance of a thorough clinical evaluation and histopathological examination to differentiate between these entities and guide appropriate management strategies.

**Figure 11:**
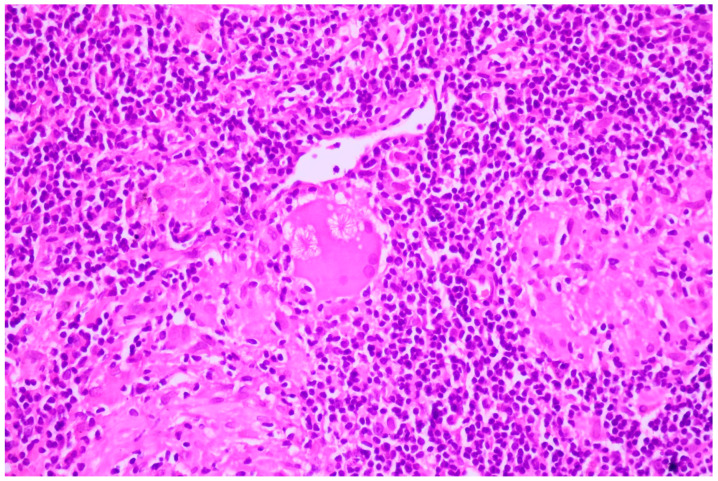
Asteroid bodies in sarcoidosis; HE, 400×.

Sarcoidosis is characterized by the presence of well-defined, compact, non-necrotizing granulomas primarily localized within the interstitium and excluding airspaces [16]. These granulomas are frequently observed in anatomical structures such as the pleura, interlobular septa, and broncho-vascular bundles, typically following lymphatic routes [16]. While a thin rim of chronic inflammation may surround these granulomas, it generally does not extend into the adjacent interstitium [16]. Although granulomas may occasionally contain small areas of central necrosis, such occurrences are typically inconspicuous [16]. Furthermore, these granulomas may harbor common multinucleated giant cells exhibiting various inclusions, including Schaumann or asteroid bodies (Figure 11) [16]. 

Large polarizable crystals of calcium oxalate and calcium carbonate, arising as byproducts of endogenous cell metabolism, can sporadically be identified within the granulomas [16]. However, it is important to note that these crystals are not specific to sarcoidosis and are commonly encountered in various chronic granulomatous conditions [16]. Care should be taken not to mistake them for exogenously administered drugs, which may be injected or inhaled [16]. Moreover, circumferential fibrosis may encase some granulomas, while others may undergo partial replacement by hyalinized fibrosis [16]. These additional alterations often coincide with the presence of granulomatous vasculitis, characterized by non-necrotizing granulomas within the intima and media of blood vessels, potentially leading to luminal constriction [16]. Importantly, this vasculitis typically occurs concurrently with or shortly after parenchymal granulomatous inflammation [16]. However, vascular necrosis is not a characteristic feature of sarcoidosis [16].

Recognizing the importance of maintaining a broad and inclusive approach to differential diagnosis is paramount, given the potential for misdiagnosis, with some cases erroneously classified as sarcoidosis instead of malignancies [14]. To accurately distinguish between sarcoidosis and sarcoid-like reactions secondary to neoplasia, a detailed histological examination is necessary [17]. Notably, cancerous cells may be embedded within a granulomatous response on the histological analysis [17]. Furthermore, lymphocyte phenotyping should be undertaken to rule out clonality in cases of atypical sarcoidosis [17]. Employing complementary immunohistochemical staining and a thorough evaluation of all included samples (which heightens the sensitivity of the histological investigation) may also be beneficial [17]. It is worth noting that certain malignancies, such as necrotic granuloma-like Hodgkin’s lymphoma and certain T-cell lymphomas (non-Hodgkin’s lymphoma), can elicit a significant tumor-related sarcoid reaction, potentially leading to its misinterpretation as non-neoplastic granulomas, such as sarcoidosis [17]. Only through comprehensive histological study can an accurate diagnosis be achieved [17]. This underscores the critical role of the histopathological analysis in elucidating the true nature of granulomatous lesions and guiding appropriate clinical management.

The association between surgically treated lung cancer and sarcoidosis is exceptionally rare, as evidenced by limited occurrences; for example, in Japan until 2003, there were only 13 occurrences [18]. Notably, there appears to be no gender imbalance in the disease, with the average patient age at diagnosis reported as 63 years [18]. Pathologically, the cases revealed a varied histological spectrum, with squamous cell carcinoma identified in four cases and adenocarcinoma in nine patients [18]. Additionally, twelve of them exhibited enlarged mediastinal and hilar lymph nodes, underscoring the potential diagnostic challenge posed by concurrent sarcoidosis and lung cancer [18].

In our study cohort, all four cases were primarily detected in the lymph nodes, with only one case additionally involving the lung parenchyma. Three of these cases were associated with tumors of neuroendocrine origin, including typical and atypical carcinoid tumors, as well as large-cell neuroendocrine carcinoma). Conversely, only one case was concomitant with squamous cell carcinoma, highlighting the diverse histological spectrum of lung cancers that may coexist.

Necrotizing granulomatous inflammation characterized by variable numbers of non-necrotizing granulomas is the hallmark histological feature in tuberculosis [16]. Typically, the necrotic areas include amorphous granular debris devoid of any discernible lung structures, albeit the fact that, occasionally, remnants of alveolar septa may be detected [16]. Granulomas often display rounded contours with well-defined margins, although irregular-shaped necrosis can sometimes happen [16]. Bronchiolar involvement by the granulomatous inflammation is common, and, infrequently, a distribution that is predominately airway-centered might occur, raising the possibility of bronchocentric granulomatosis as a differential diagnosis [16,19].

In our study, tuberculosis was identified in five cases, with four cases affecting the lung parenchyma and one case involving both the lung parenchyma and the lymph nodes. The results are consistent with other reported data in the literature where parenchymatous tuberculosis was more frequently associated with lung cancer than the endobronchial one [20]. This observation underscores the potential significance of parenchymal tuberculosis as a predisposing factor or coexisting condition in patients with lung cancer.

In contrast to our study’s findings, which revealed cases of tuberculosis coexisting with various types of non-small-cell lung cancer (NSCLC), including adenocarcinoma, large-cell neuroendocrine carcinoma, and combined histologies (squamous cell carcinoma combined with small-cell carcinoma), other investigations have reported a predominance of squamous cell carcinoma among lung cancer cases associated with pulmonary tuberculosis. For instance, in a study encompassing 374 patients with pulmonary tuberculosis, of which 16 also had lung cancer, the most frequent type of cancer was squamous cell carcinoma [21]. This discrepancy underscores the diverse histopathological landscape and the potential interplay between tuberculosis and different subtypes of lung cancer, necessitating a further exploration into the underlying mechanisms and clinical implications of these associations.

The presence of nodular calcification in lung lesions can pose diagnosis difficulties during imagistic examinations, as they are identified in multiple types of lung lesions [22,23]. Unlike most primary lung malignancies, including small cell, squamous cell, adenocarcinoma, carcinoid, and mucoepidermoid carcinomas, which rarely exhibit calcifications within a lung lesion, nodular calcifications are commonly associated with benign processes [24]. Moreover, their association with lung cancer may lead to an incorrect diagnosis [22]. This should serve as a caution that calcifications are not necessarily a benign process [24]. Infections, lung metastases, chronic pulmonary bleeding, pneumoconiosis, deposition illnesses, and idiopathic conditions like pulmonary alveolar microlithiasis are among the conditions that can cause diffusely distributed smaller calcified nodules [13,22]. Additionally, calcified lesions may also include *Histoplasma capsulatum*, *Coccidioides immitis*, tuberculosis, lung cancer, condrosarcoma, osteosarcoma, or others [22,25]. This underscores the necessity for a comprehensive evaluation and correlation with clinical and pathological findings to accurately determine the underlying etiology of calcified nodules in the lungs.

Osseous stromal metaplasia, although rare, has been described in the literature for both benign and malignant neoplasms [26]. Although multiple cases have not been reported, it is interesting that, in our study, we have four cases of osseous metaplasia. This observation adds to the existing body of knowledge and underscores the importance of recognizing and documenting such occurrences, as they contribute to our understanding of the histopathological diversity and complexity of neoplastic processes in the lungs. Pneumoconiosis is typically caused by inhaling inorganic dust particles in occupational settings, eliciting a fibrotic response in the lungs [12,27]. However, there are notable exceptions to this pattern, with certain cases developing over shorter durations. Pathologists must be vigilant regarding the diagnostic features of these conditions, as the pathological findings can resemble those of other fibrotic and granulomatous lung disorders [27]. Pneumoconiosis was associated with three cases of lung cancer from our study group, all exhibiting mild forms. Similarly, in other studies, it was noticed that the patients who had lung cancer tended to present with milder forms of pneumoconiosis, compared to the ones with only pneumoconiosis who had a more severe and complicated pneumoconiosis [28]. It is possible that the cases of pneumoconiosis associated with lung cancer in our study were in a mild form because they were diagnosed earlier when examined for lung cancer. In a study including 563 patients with pneumoconiosis, for the ones with associated cancer, it was not possible to link occupational exposure to cancer [28]. But, nevertheless, in our study group, the coexistence of cancer and pneumoconiosis may have had a common cause, since some causes of pneumoconiosis may be involved in carcinogenesis as well; for example, crystalline silica dust has been identified as carcinogen group I [29]. Some studies suggested that different types of miRNA involved in pneumoconiosis, for example, miR-125a, can also contribute to cancer development [29]. These findings highlight the intricate interplay between occupational exposures, molecular mechanisms, and disease pathogenesis, underscoring the need for further research to elucidate these complex relationships.

**Figure 12:**
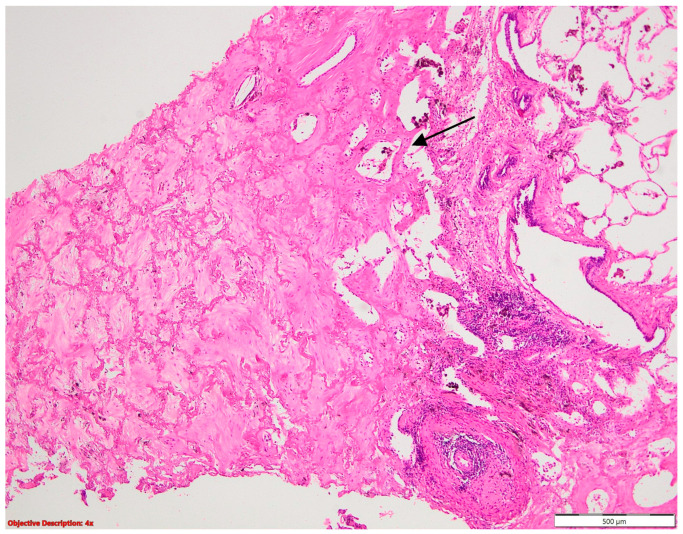
Lung infarction (→): necrotic alveoli are evident on the left side of the picture; HE, 40×.

Regarding NSLSC associated with lung infarction (Figure 12), we had four patients in our study, but only for two patients was the lymphovascular invasion microscopically identified. This is a limit of the pathological examination, given the fact that these patients may, in fact, have neoplastic emboli, but these may have not been noticed on the examined slides. Another possible cause of lung infarction may be thrombosis, in the context of neoplastic disease. A Dutch study performed over a period of 7 years, including 3717 patients with lung cancer, highlighted that lung cancer patients had a 6-fold higher likelihood than their cancer-free controls of being hospitalized for pulmonary embolism in the 12 months preceding lung cancer diagnosis [30]. Following diagnosis, lung cancer patients had a 5-fold higher risk of pulmonary embolism in the following 6 months and a 17-fold higher risk in the first 6 months [30]. However, when compared to pulmonary embolism in people without cancer, those with cancer often had less aggressive early clinical symptoms and thrombus burden [31]. These findings highlight the intricate relationship between lung cancer and pulmonary embolism, emphasizing the need for heightened vigilance in identifying and managing thrombotic complications in patients with neoplastic disease. Additionally, they underscore the importance of considering thrombosis as a potential cause of lung infarction in the context of lung cancer.

**Figure 13:**
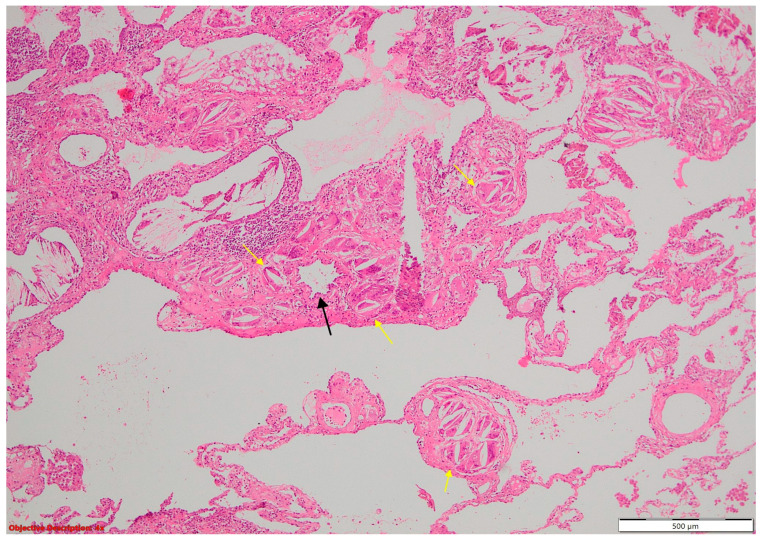
Aspiration pneumonia. Multiple histiocytes with intracytoplasmic cholesterol (**→**) are present in the peribronchiolar area (→); HE, 40×.

Aspiration pneumonia (Figure 13) is often associated with glottal incompetence and ciliary dysfunction, which can result from radiation therapy [32]. The tumor invasion of the vagus or recurrent laryngeal nerve is a known complication in individuals with lung cancer [32]. All these may cause one to be predisposed to aspiration pneumonia in lung cancer. In our study, we identified only one case of lung cancer, which underscores the relatively rare occurrence of this complication in our cohort. Nonetheless, the presence of lung cancer, particularly in conjunction with predisposing factors such as glottal incompetence and nerve invasion, heightens the risk of aspiration pneumonia. This highlights the importance of vigilance in identifying and managing such complications in patients with lung cancer, particularly those undergoing radiation therapy or presenting with neurological symptoms suggestive of nerve involvement.

**Figure 14:**
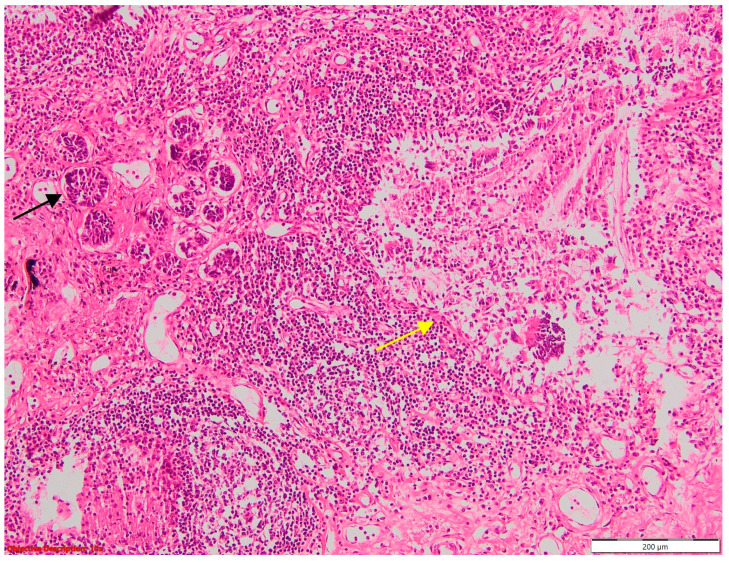
Tumorlet—clusters of neuroendocrine cells (→) are present adjacent to a bronchiolar structure, with detached epithelium (**→**); significant inflammatory infiltrate; HE, 100×.

**Figure 15:**
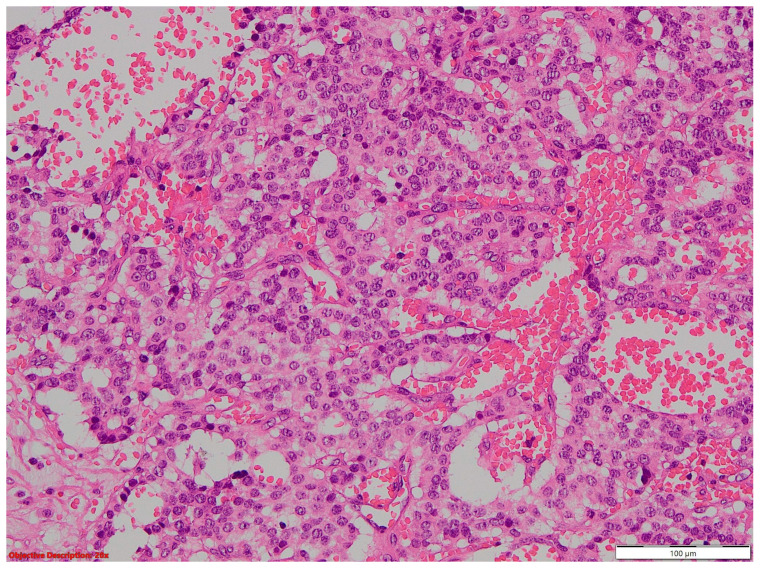
Typical carcinoid: proliferation of neuroendocrine cells, without necrosis and/or mitotic activity; HE, 200×.

Tumorlets (Figure 14) were associated with adenocarcinoma in one case and another case with typical carcinoid (Figure 15) and sarcoidosis.

Women in their middle years, between 50 and 70, who have previously been diagnosed with cancer, particularly breast carcinoma, are more likely to develop tumorlets [33,34]. Even though they are surrounded by inflammatory and reactive tissue and fibrosis, tumorlets are typically asymptomatic and show no signs of severely restrictive or obstructive lung disease, even when they are numerous [33,34]. This highlights the indolent nature of tumorlets and underscores the importance of vigilant surveillance and management strategies, particularly in individuals with a history of cancer.

The most frequent benign lung tumor is hamartoma, composed of tissues that are commonly found in the lung, such as cartilage, fat, and epithelial and fibrous tissue [22]. Despite their benign nature, hamartomas exhibit chaotic growth patterns [22], and are characterized by distinctive popcorn calcifications and fat attenuation [24]. The majority of pulmonary hamartomas are asymptomatic and do not have any malignant potential [22]. The differential diagnosis must be made from other fat-containing lesions, for example, germ cell tumors, lipomas, liposarcomas, and thymolipoma [22]. Given their prevalence, it is not surprising that both cases of benign tumors associated with lung cancer in our study were represented by hamartomas. This underscores the importance of an accurate diagnosis and the recognition of common benign lesions in lung cancer patients.

The limitations of our study are inherent in the retrospective design (potential biases in patient selection, data collection, and interpretation) and the development of the study at a single medical institution (Marius Nasta Institute of Pneumophthisiology, Bucharest, Romania). This may limit the generalizability of the findings to other settings or populations. Different institutions and geographic locations may have varying patient demographics, treatment approaches, and prevalence of comorbid conditions. 

We focused our study only on patients with lung tumor types for which surgical resection is a viable therapeutic option such as carcinoids and NSCLC lesions, representing the majority of lung tumors. Given that the data were collected from patients diagnosed in 2019, it is pertinent to consider the potential impact of subsequent advancements in diagnostic technologies, treatment protocols, and healthcare practices on the applicability of our findings to current clinical settings. For instance, the introduction of next-generation sequencing techniques may have led to theenhanced molecular profiling of NSCLC tumors, potentially influencing treatment selection based on actionable genetic alterations. Moreover, the emergence of immune checkpoint inhibitors as standard-of-care therapies and the refinement of patient stratification algorithms may have altered the therapeutic landscape since the time of data collection. Additionally, changes in healthcare delivery models, such as the implementation of telemedicine and multidisciplinary care pathways, could have influenced the diagnostic efficiency and treatment decision-making processes. Therefore, while our findings provide valuable insights into carcinoid and NSCLC complexities, their interpretation should be tempered by an awareness of the evolving clinical context and contemporary standards of care. However, all of these did not affect the relevance or applicability of the findings to current practices. It is still crucial that we establish the tumoral stage since it is a major factor in the treatment.

Given the limitations of our article, we consider that further research is needed, including a study of different populations, employing longitudinal designs, or using multicentric data to confirm our findings or to highlight possible differences depending on the geographical area.

Most of the included cases went through lobectomy and none was exclusively an atypical resection. Moreover, some of them went through a whole-lung resection. Therefore, the surgery specimens were diverse and there were no statistically significant results in our study group; this is the reason for not including these data in the article. We acknowledge that this aspect could bear heavily on the interpretation of our results, particularly concerning lesion localization and staging. Future prospective studies are warranted to collect comprehensive data on surgical interventions in carcinoid and NSCLC cases.

## 5. Conclusions

There were no significant differences between patients presenting only malignant lesions and those with coexisting nonmalignant lesions in terms of demographic and exposure profiles, emphasizing the difficulty of reaching a precise diagnosis and accurate staging using only clinical/imagistics data.

Concomitant non-malign pulmonary lesions may lead to the over-staging of lung cancer. This seems particularly relevant in high-prevalence tuberculosis regions where pulmonary and mediastinal involvement should be expected to frequently occur. Such data suggest an increasingly important role in pathology staging. 

These findings underscore the complexities involved in diagnosing and staging lung cancer, highlighting the pivotal role of a histopathological examination in guiding appropriate diagnostic and therapeutic strategies for patients with associated lesions.

## Figures and Tables

**Table 1:** Association and localization of coexisting lesions.

Case Number	Lesion 1	Lesion 2	Lesion 3
1	Atypical carcinoid (RUL)	Sarcoidosis (lymph node)	
2	ADC (LUL)	Hamartoma (LIS)	
3	ADC (RUL)	SqCC (LSD)	Fibronodular lesion (lung)
4	SqCC (left main bronchus)	Fibronodular lesion (lymph node)	
5	SqCC (LUL)	Fibronecrotic nodule (lung)	
6	Typical carcinoid (ML)	Sarcoidosis (lung + lymph node)	Tumorlet
7	ADC (LLL)	Hamartoma	
8	ADC (RUL)	Fibronodular lesion (lung)	Osseous metaplasia
9	ADC (RUL)	TBC (lung)	
10	ADC (RLL)	Fibronodular lesion (lung)	
11	ADC (LLL)	TBC (lung)	
12	LCNEC (RLL)	Sarcoidosis (lymph node)	
13	ADC (ML)	Fibronecrotic nodule (lymph node)	
14	SqCC (RLL)	Fibronecrotic nodule (lung)	
15	ADC (RUL)	Fibronodular lesion (lung)	Osseous metaplasia
16	ADC (LLL)	Meningothelial-like nodule	Calcified nodule
17	ADC (RUL)	Fibronodular lesion (lung)	
18	ADC (LUL)	Lung infarction	
19	ADC (RUL)	Fibronodular lesion (lymph node)	
20	ADC (RUL)	Fibronecrotic nodule (lung)	Calcified nodule
21	Combined carcinoma (SqCC + SCLC) (RUL)	TBC (lung)	
22	SqCC (RLL)	Lung infarction	
23	SqCC (RUL)	Osteo medullary metaplasia	
24	SqCC (LUL)	Fibronecrotic nodule (lung)	
25	ADC (RLL)	Lung infarction	
26	Pleomorphic carcinoma (ADC component) (RLL)	Fibronodular lesion (lung)	
27	ADC (LUL)	Fibronodular lesion (lymph node)	Tumorlet
28	ADC (LLL)	Fibronodular lesion (lung)	
29	Pleomorphic carcinoma (RUL)	Lung infarction	Aspiration pneumonia
30	NSCLC (RUL)	TBC (lung)	
31	ADC (RUL)	Fibronodular lesion (lung)	
32	LCNEC (RUL)	TBC (lung + lymph node)	
33	ADC (ML)	LCNEC (2 tumors; ML)	Fibronodular lesion (lung)
34	SqCC (RUL)	Sarcoidosis (lymph node)	
35	ADC (RUL)	AAH	
36	ADC (RUL)	Fibronodular lesion (lung)	Osteomedullary metaplasia
37	ADC (RUL)	ADC MIA (ML)	AAH

ADC—invasive adenocarcinoma; ADC MIA—minimally invasive adenocarcinoma; SqCC—invasive squamous cell carcinoma; LCNEC—large cell neuroendocrine carcinoma; SCLC—small-cell neuroendocrine carcinoma; NSCLC—non-small-cell carcinoma; AAH—atypical adenomatous hyperplasia; TBC—tuberculosis; RUL—right upper lobe; LUL—left upper lobe; ML—middle lobe; LLL—left lower lobe; RLL—right lower lobe.

## Data Availability

Data are contained within the article.
